# Effect of switching from acenocoumarol to phenprocoumon on time in therapeutic range and INR variability: A cohort study

**DOI:** 10.1371/journal.pone.0235639

**Published:** 2020-07-10

**Authors:** Jasper H. A. van Miert, Nic J. G. M. Veeger, Arina J. ten Cate-Hoek, Margriet Piersma-Wichers, Karina Meijer

**Affiliations:** 1 Department of Hematology, University of Groningen, University Medical Center Groningen, Groningen, the Netherlands; 2 Certe Thrombosis Service Groningen, Groningen, the Netherlands; 3 Department of Epidemiology, University of Groningen, University Medical Center Groningen, Groningen, the Netherlands; 4 Department of Vascular Medicine, Heart and Vascular Center, Maastricht University Medical Centre, Maastricht, the Netherlands; 5 Laboratory for Thrombosis and Hemostasis, Cardiovascular Research Institute, Maastricht, the Netherlands; Hospital Dr. Rafael A. Calderón Guardia, CCSS, COSTA RICA

## Abstract

**Background:**

Treatment with vitamin K antagonists (VKA) requires a high proportion of time in the therapeutic range (TTR) and a low international normalised ratio (INR) variability to be maximally safe and effective. Switching from short-acting acenocoumarol to long-acting phenprocoumon could improve VKA control.

**Aims:**

We assessed whether switching from acenocoumarol to phenprocoumon improves the time in the therapeutic range (TTR) and INR variability.

**Methods and results:**

In a retrospective cohort with data on 236,957 patients-years of VKA management from two first-line anticoagulation clinics in the Netherlands, we identified 124 patients in target range 2–3, 269 patients in target range 2–3.5 and 98 patients in target range 2.5–3.5 who switched from acenocoumarol to phenprocoumon. They were matched in a 1:2 ratio to non-switching controls using propensity score matching. Over the first 180 days after a switch, switchers’ TTR declined 5 (95% CI 1 to 10), 10 (95% CI 7 to 13) and 5 (95% CI 0 to 11) percentage points relative to non-switchers, in target ranges 2–3, 2–3.5 and 2.5–3.5. Anticoagulation was more often supra-therapeutic in switchers, and switchers had a higher INR variability. In the following 180 days, TTR in switchers became 1 (95% CI -4 to 6), 4 (95% CI 0 to 7) and 6 (95% CI 1 to 12) percentage points better than in non-switchers. Switchers’ INRs were much more stable than non-switchers’.

**Conclusion:**

Eventually, a switch from acenocoumarol to phenprocoumon leads to a higher TTR and a lower INR variability. However, this is preceded by a transition period with opposite effects. An improved conversion algorithm could possibly shorten the transition period. Until then, physicians and patients should decide whether switching is worth the increased risk during the transition phase.

## Introduction

Vitamin K antagonists (VKAs) are prescribed to treat and prevent thrombosis in venous thromboembolism, atrial fibrillation, heart valve replacement, and some other indications. Their effect is unpredictable and requires frequent monitoring to obtain an anticoagulation intensity (international normalised ratio (INR)) within the therapeutic range. Treatment is safest and most effective when the time within the therapeutic range (TTR) is high [1,2] and the INR variability is low [3]. Unfortunately, despite frequent monitoring, not all patients achieve this.

With the introduction of the direct oral anticoagulants (DOACs), the treatment arsenal expanded. Many patients have switched from a VKA to a DOAC. However, DOACs are contraindicated in mechanical heart valves, renal insufficiency, and in combination with certain interacting drugs. Furthermore, because a poor TTR can be the result of poor medication adherence, physicians may be reluctant to switch such patients to a DOAC. Therefore, interventions to optimise VKA treatment remain relevant.

A possible intervention is to switch to a vitamin K antagonist with a longer half-life. Theoretically, a longer-acting drug would be less sensitive to suboptimal medication adherence and produce more stable INRs. In the Netherlands, only short-acting acenocoumarol (half-life 8–11 hours) and long-acting phenprocoumon (half-life 160 hours) are available. Data on the effect of switching from acenocoumarol to phenprocoumon on TTR are lacking completely; studies with other VKAs ignore natural variation in TTR. We aimed to determine whether switching from acenocoumarol to phenprocoumon increased TTR and decreased INR variability in practice.

## Methods

This is a retrospective cohort study with patients from two first-line thrombosis services in the Netherlands. Of patients who switched to phenprocoumon, we compared TTR and INR variability before and after the switch. We also assessed whether this change differed from that in matched non-switchers, to account for the natural evolution of TTR and INR variability over time. We hypothesised that the switch to phenprocoumon would increase TTR and decrease INR variability.

### Data sources and patient selection

In the Netherlands, VKA management is delegated to dedicated first-line regional “Thrombosis Services” that organise INR checks and adjust the VKA dose. Each thrombosis service uses one preferred VKA. Certe Trombosedienst and Trombosedienst Maastricht UMC+ are two thrombosis services, in the north and south of the Netherlands, respectively, that routinely use acenocoumarol. Thrombosis Service physicians can suggest switching a patient to phenprocoumon; the treating physicians usually follow this suggestion.

We extracted INR and VKA dosing records of adult patients who used acenocoumarol or switched from acenocoumarol to phenprocoumon, in target ranges 2–3, 2.5–3.5 or 2–3.5 (used for low-intensity anticoagulation before switching to the internationally recommended target range of 2–3 on January 1st, 2016), indicated for atrial fibrillation (AF), venous thromboembolism (VTE) or mechanical heart valve replacement (MVR). Data were extracted on July 17th, 2018 (Certe Trombosedienst) and June 20th, 2019 (Trombosedienst Maastricht UMC+). We excluded the first 90 days of treatment with vitamin K antagonists because INRs are unstable in the initiation period.

All data were anonymised before they were provided to the researchers. The Medical Ethics Review Board of the University of Groningen confirmed that this study required no ethical approval.

#### Switchers

We selected patients who used acenocoumarol for at least 180 days (excluding the initial 90 days) before switching to phenprocoumon, and had follow-up INR measurements for at least a further 180 days. The study size was based on the number of patients who switched.

#### Non-switchers

Non-switchers were defined as patients who used acenocoumarol during their entire treatment course or up to date of data extraction. For every calendar date on which a switcher switched, we selected patients who used acenocoumarol for the previous 180 days (excluding the initial 90 days) and next 180 days, with the same target range. At this stage, one non-switcher could be selected to be a control for multiple switch dates, if their follow-up was long enough. We then later randomly selected one instance per patient, to prevent over-representation of long-term non-switchers in the matching strategies.

#### Subgroups

We defined subgroups of special interest, based on the prompt for switching and special clinical considerations. A switch could be prompted by a low acenocoumarol dose requirement, a suboptimal TTR or high variability. We separately analysed patients who used less than 1.5mg acenocoumarol per day, patients who had a TTR <60% and patients with a baseline INR variability above the 75th percentile in their target range. In addition, we provide specific information about patients with a mechanical heart valve (who are confined to VKA), and the elderly (aged ≥70 years; who are more likely to have contraindications to restrict other forms of anticoagulation).

### Outcomes

We calculated the proportion of time in the therapeutic range (TTR) as well as the time below and above the range for each patient using linear interpolation according to the Rosendaal method [4]. Additionally, TTR was dichotomised with a cut-off of 65% because patients with a TTR ≥ 65% have a favourable risk profile for bleeding and thrombosis [1]. INR variability was calculated using Fihn’s method [5] (equation). Furthermore, we determined the mean INR (without interpolation), the mean time between INR measurements, and the mean VKA dose in milligrams.

$$\mathrm{INR}\;\mathrm{variability}=\sqrt{\frac{1}{n-1}\sum_{i=2}^{n}\frac{({\mathrm{INR}}_{i}-{\mathrm{INR}}_{i-1}{)}^{2}}{\Delta t_{\left(\mathrm{days}\right)}}}$$

**Fig 1:**
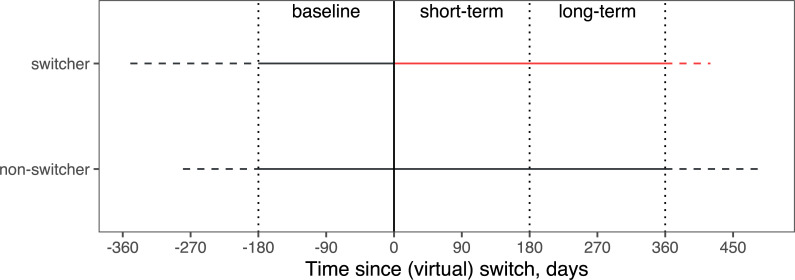
Periods distinguished in the analyses. Solid lines indicate the time period used in the analyses. A period in which acenocoumarol was used, is shown in black; phenprocoumon is shown in red.

We distinguished three periods: the 180 days before a patient’s switch to phenprocoumon, the 180 days after switch, and a period from 180 to 360 days (see Fig 1). For non-switchers, we used the switch date from the corresponding switcher. All differences were calculated with the 180 days before the switch (baseline) as reference. Because the distribution of the INR variability is skewed, the difference of the log-transformed INR variability was used (which is equivalent to the log-transformed ratio of INR variabilities). The VKA dose is reported as the mean dose during a certain period, and as a percentage of a patient’s mean dose during the 180 days before the switch.

### Statistical analyses

#### Switchers over time

For the initial analysis, we summarised the TTR and INR variability as median and interquartile range. Differences were tested using a one-sample Wilcoxon rank-sum test. Intra-individual correlations were summarised using the Spearman correlation.

#### Switchers compared with non-switchers

We compared the differences in VKA quality parameters between switchers and non-switchers, to isolate the effect of the switch to phenprocoumon. Non-switchers were matched to switchers in a 2:1 ratio using propensity scores based on age, sex, indication for VKA therapy, duration of VKA therapy until (virtual) switch, the mean acenocoumarol dose in the six months before (virtual) switch, the times below and above range and the log-transformed INR variability. The propensity score was determined by logistic regression, and matching was performed using the MatchIt package [6] in R version 3.6.1 (2019-07-05) (R core team, Vienna, Austria). This matching procedure was repeated for every group of special interest.

Differences from baseline were analysed using a mixed linear effects model [7] to account for non-independence within matched sets. The time between INR measurements was compared in the later periods independent from the time between INR measurements during baseline, to prevent distortion by any extra monitoring before the anticipated switch. The time between INR measurements was log-transformed to approach normality. We calculated the difference in probability to obtain a good TTR (≥ 65%) between switchers and matched non-switchers based on the observed frequencies.

#### Reporting

Values are reported as mean ± SD or median [interquartile range], as appropriate. A p-value below 5% was considered significant for all analyses; estimates are given as point estimate (95% confidence interval).

## Results

### Patient selection

**Fig 2:**
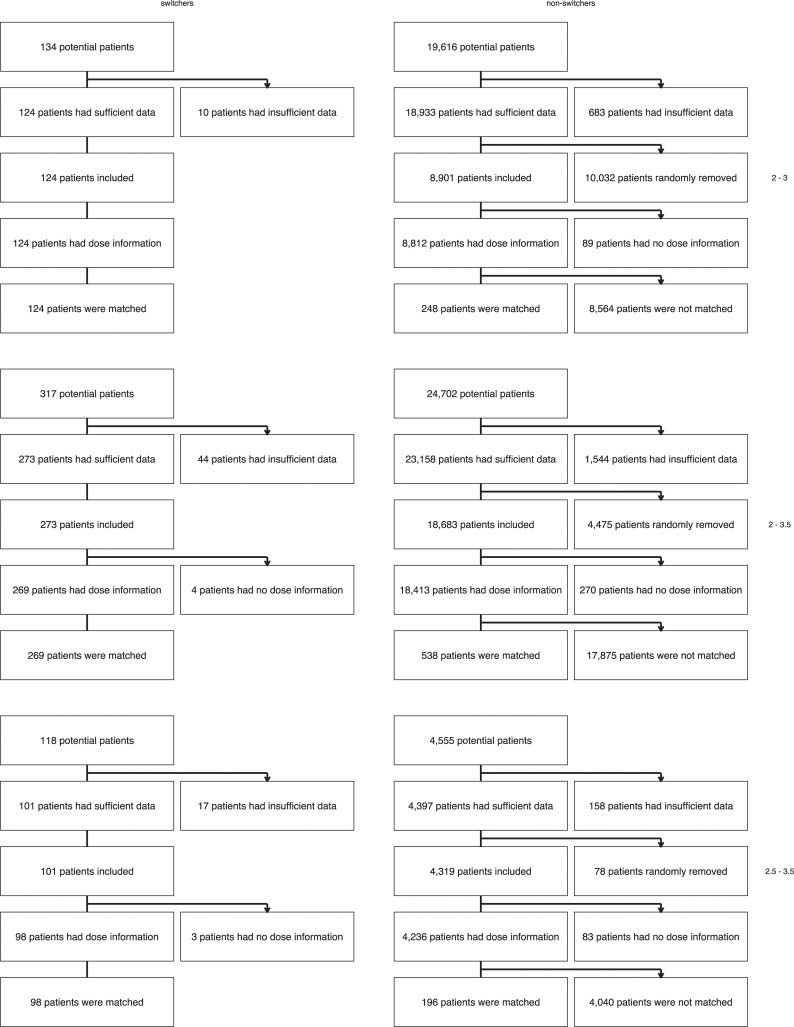
Flowchart of patient selection in this study.

Patient selection is outlined in Fig 2.

The thrombosis service Groningen contributed 28,756 eligible patients from September 25, 1991, onwards, with a total follow-up of 207,963 patient-years. The thrombosis service Maastricht contributed 3,645 patients from January 02, 1997, onwards, with 28,994 patient-years of follow-up.

There were relatively more switchers in Maastricht than in Groningen. Although Maastricht contributed only 11% of patients, they contributed 47% of switchers (234, versus 264 in Groningen). Four hundred and ninety-one switchers could be matched to 982 controls.

### Patient characteristics

Characteristics of switchers are summarised in Table 1; characteristics of all potential controls, and subgroups of switchers, are shown in the Supplementary Material.

**Table 1:** Characteristics of patients included in the study.

Target range	2.0–3.0	2.0–3.5	2.5–3.5
N	124	273	101
Center Groningen	30	166	68
Center Maastricht	94	107	33
Age (median [IQR])	80 [68, 85]	73 [62, 80]	65 [56, 74]
Male gender (%)	56 (45.2)	123 (45.1)	55 (54.5)
VKA experience (median [IQR])	5 [3, 11]	2 [1, 5]	6 [2, 12]
Dose (median [IQR])	1.1 [0.9, 2.8]	2.7 [1.5, 4.3]	3.1 [1.8, 4.3]
Acenocoumarol dose <1.5mg (%)	75 (60.5)	68 (25.3)	21 (21.4)
Below range (median [IQR])	23.8 [15.5, 37.7]	13.8 [4.4, 27.6]	28.7 [10.5, 49.2]
TTR (median [IQR])	45.3 [33.6, 53.7]	64.1 [47.0, 79.0]	36.5 [21.5, 47.5]
Above range (median [IQR])	27.9 [15.7, 40.5]	14.4 [2.2, 29.8]	32.0 [13.3, 46.4]
TTR <60% (%)	106 (85.5)	119 (43.6)	89 (88.1)
Mean INR (median [IQR])	2.7 [2.3, 2.9]	2.8 [2.4, 3.2]	3.2 [2.6, 3.6]
INR variability (median [IQR])	0.52 [0.32, 0.80]	0.40 [0.23, 0.67]	0.50 [0.31, 0.91]
Mean number of days between INRs (median [IQR])	11.7 [9.6, 14.6]	14.0 [10.7, 17.6]	11.9 [9.2, 14.6]
Atrial fibrillation (%)	96 (77.4)	223 (81.7)	43 (42.6)
Venous thromboembolism (%)	27 (21.8)	52 (19.0)	12 (11.9)
Mechanical heart valve (%)	6 (4.8)	5 (1.8)	64 (63.4)

Switchers achieved a lower TTR and their INRs were more volatile in the period before they switched than unmatched non-switchers.

The daily acenocoumarol dose differed between cases and controls as well: switchers from Maastricht, where a low dose requirement is a trigger to consider switching, indeed required a lower dose than unmatched non-switchers. In Groningen, where no such trigger is formally defined, switchers generally use a higher dose than the other patients in the anticoagulation clinic.

Men are less likely to be switched across all target ranges, yet they still outnumber women in target range 2.5–3.5, as they are more prone to cardiac indications for anticoagulation.

### Switching: Dosing and monitoring

Patients who switched from acenocoumarol to phenprocoumon were given a lower VKA dose in milligrams. For target range 2–3, the phenprocoumon dose was lowered to 91 [78–101]% of the acenocoumarol dose (in mg) in the first 180 days. In the other target ranges, the conversion factors were lower: 86 [75–97]% for target range 2–3.5 and 88 [73–96]% for target range 2.5–3.5.

Patients in target ranges 2.0–3.5 and 2.5–3.5 were monitored more often than before their switch; this was not observed in patients in target range 2.0–3.0 (Table 2). Patients who required more INR measurements before their switch, were checked more often after their switch (Spearman ρ 0.35 (95% CI 0.19 to 0.50), 0.46 (95% CI 0.36 to 0.55) and 0.54 (95% CI 0.38 to 0.66) for target ranges 2.0–3.0, 2.0–3.5 and 2.5–3.5).

**Table 2:** A. VKA quality parameters in patients in target range 2.0–3.0 who switched from acenocoumarol to phenprocoumon. **B.** VKA quality parameters in patients in target range 2.0–3.5 who switched from acenocoumarol to phenprocoumon. **C.** VKA quality parameters in patients in target range 2.5–3.5 who switched from acenocoumarol to phenprocoumon.

	Before switch	Short-term	Long-term
A			
TTR	45% [34–54]	51% [37–64], p = 0.001	62% [43–75], p = <0.001
above range	28% [16–40]	24% [12–39], p = 0.589	17% [0–36], p = 0.045
below range	24% [15–38]	18% [9–33], p = 0.011	11% [0–27], p = <0.001
INR variability	0.52 [0.32–0.80]	0.28 [0.19–0.44], p = <0.001	0.17 [0.12–0.26], p = <0.001
mean INR	2.7 [2.3–2.9]	2.6 [2.3–3.0], p = 0.773	2.5 [2.3–2.9], p = 0.385
dose	1.1 [0.9–2.8]	1.1 [0.8–2.3], p = <0.001	1.1 [0.8–2.2], p = <0.001
dose ratio	ref	91% [78–101], p = <0.001	85% [70–99], p = <0.001
mean number of days between INRs	12 [10–15]	11 [10–14], p = 0.401	17 [13–20], p = <0.001
B			
TTR	64% [47–79]	62% [48–73], p = 0.173	77% [60–92], p = <0.001
above range	14% [2–30]	25% [16–38], p = <0.001	15% [0–26], p = 0.850
below range	14% [5–28]	7% [2–17], p = <0.001	0% [0–12], p = <0.001
INR variability	0.40 [0.23–0.67]	0.32 [0.22–0.50], p = <0.001	0.18 [0.12–0.32], p = <0.001
mean INR	2.8 [2.4–3.2]	3.0 [2.7–3.4], p = <0.001	3.0 [2.7–3.2], p = <0.001
dose	2.7 [1.5–4.3]	2.3 [1.5–3.3], p = <0.001	2.0 [1.2–3.1], p = <0.001
dose ratio	ref	86% [75–97], p = <0.001	78% [67–92], p = <0.001
mean number of days between INRs	14 [11–18]	12 [10–15], p = <0.001	19 [14–27], p = <0.001
C			
TTR	36% [22–47]	36% [24–51], p = 0.684	49% [34–66], p = <0.001
above range	32% [13–47]	38% [22–56], p = 0.003	30% [7–43], p = 0.774
below range	28% [10–49]	12% [5–34], p = <0.001	10% [0–27], p = <0.001
INR variability	0.50 [0.31–0.93]	0.45 [0.31–0.61], p = 0.037	0.22 [0.14–0.43], p = <0.001
mean INR	3.2 [2.7–3.6]	3.3 [2.9–3.8], p = 0.012	3.2 [2.9–3.5], p = 0.804
dose	3.1 [1.8–4.3]	2.4 [1.6–3.5], p = <0.001	2.2 [1.6–3.2], p = <0.001
dose ratio	ref	88% [73–96], p = <0.001	79% [66–93], p = <0.001
mean number of days between INRs	12 [9–15]	11 [9–13], p = 0.013	16 [12–20], p = <0.001

P-values from Wilcoxon rank-sum test.

After the first 180 days, the phenprocoumon dose was lowered further, to 85 [71–99]% of the baseline acenocoumarol dose in target range 2–3, 78 [67–92]% in target range 2–3.5 and 79 [66–94]% in target range 2.5–3.5.

Monitoring intervals were prolonged across all target ranges (Table 2). For target range 2–3, the monitoring interval was no longer related to the monitoring interval at baseline (ρ 0.08 (95% CI -0.11 to 0.25)).

### Effect of switching

The evolution of VKA quality parameters after switching from acenocoumarol to phenprocoumon is summarised in Table 2A–2C. Patients in target range 2–3 showed a marked immediate improvement in TTR. This was not true for patients in the other target ranges, where the improvements were only seen in the long-term period (180 to 360 days after the switch). In these target ranges, the time above the target range increased at the expense of not only the time below the range, but also the time in the therapeutic range. INR variability decreased across all target ranges.

The TTR achieved after the switch was only weakly related to the TTR before the switch: Spearman ρ was 0.26 (95% CI 0.09 to 0.42) for target range 2.0–3.0, 0.22 (95% CI 0.10 to 0.33) for target range 2.0–3.5 and 0.05 (95% CI -0.15 to 0.24) for target range 2.5–3.5. In target range 2.5–3.5 there was a moderate positive association between the time above the range at baseline and after the switch (ρ 0.43 (95% CI 0.26 to 0.58)), that persisted in the later period (ρ 0.45 (95% CI 0.27 to 0.59)). The same was found for the time below the range (ρ 0.41 (95% CI 0.24 to 0.56) and 0.37 (95% CI 0.19 to 0.53)). In other target ranges, this effect was not observed.

### Comparison with non-switchers

**Fig 3:**
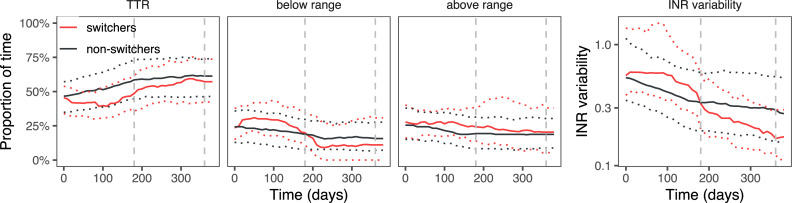
Proportion of time spent in, above or below the target range of 2.0–3.0, and INR variability, in switchers and matched non-switchers. Median and interquartile range.

**Fig 4:**
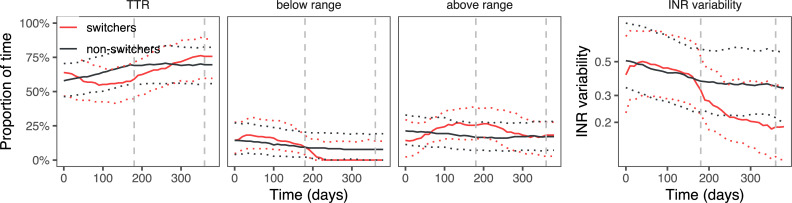
Proportion of time spent in, above or below the target range of 2.0–3.5, and INR variability, in switchers and matched non-switchers. Median and interquartile range.

**Fig 5:**
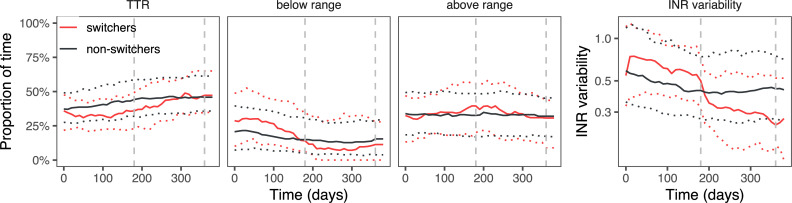
Proportion of time spent in, above or below the target range of 2.5–3.5, and INR variability, in switchers and matched non-switchers. Median and interquartile range.

Four hundred and ninety-one switchers could be matched with 982 non-switching controls, with a high similarity between the two groups (standardised mean difference in propensity scores was 0.08 for target range 2–3; 0.02 for target range 2–3.5 and 0.07 for target range 2.5–3.5). The coefficients from the propensity score matching and the patient characteristics from matched patients are shown in S3 and S4 Tables, respectively. The difference in outcomes between switchers and matched non-switchers is shown in Table 3A–3C and Figs 3–5.

When comparing switchers with non-switchers, the initial effect of switching is negative: TTR decreases mainly owing to an increase in the time above the range. At the same time, the INR variability increases, though only statistically significantly in target range 2–3.5. In the first six months, switchers are less likely to obtain or maintain a TTR ≥65%: the absolute risk difference was -13 (95% CI -22 to -3) percentage points for target range 2.0–3.0; for target range 2.0–3.5 it was -20 (95% CI -27 to -12); for target range 2.5–3.5 the risk difference was -5 (95% CI -12 to 3). Switchers’ INRs were monitored more often, with 24% (95% CI 18 to 29) less time between INR measurements for target range 2–3; 22% (95% CI 18 to 25) for target range 2–3.5 and 29% (95% CI 23 to 35) for target range 2.5–3.5.

Positive effects occur in the second six-month period after switching. TTR mildly increases, with 0.8 (95% CI -4.4 to 6.1), 3.9 (95% CI 0.4 to 7.4) and 6.4 (95% CI 0.8 to 12.1) percentage points in target ranges 2.0–3.0, 2.0–3.5 and 2.5–3.5, respectively. The INR variability strongly decreases across all target ranges. During this period, switchers in target ranges 2–3 and 2–3.5 were slightly more likely to obtain a TTR ≥65%: absolute risk differences were 2 (95% CI -9 to 13) and 6 (95% CI -1 to 13). The effect was more pronounced in target range 2.5–3.5, where patients were 15 (95% CI 6 to 25) percentage points more likely to obtain a TTR ≥65%. Switchers in target ranges 2–3 and 2.5–3.5 were no longer monitored more often than their matched non-switching controls (they had 2% (95% CI -6 to 12) and 2% (95% CI -6 to 12) more time between measurements). Patients in target range 2–3.5 had 14% (95% CI 8 to 22) more time between INR measurements.

**Table 3:** A. Difference in changes since baseline of switchers versus matched non-switching controls in target range 2.0–3.0, with 95% confidence interval. **B.** Difference in changes since baseline of switchers versus matched non-switching controls in target range 2.0–3.5, with 95% confidence interval. **C.** Difference in changes since baseline of switchers versus matched non-switching controls in target range 2.5–3.5, with 95% confidence interval.

	Short-term difference	Long-term difference
A		
TTR	-5.5pp (-9.6 to -1.3), p = 0.01	0.8pp (-4.4 to 6.1), p = 0.76
above range	5.8pp (0.8 to 10.8), p = 0.02	0.8pp (-4.9 to 6.4), p = 0.79
below range	-0.3pp (-4.9 to 4.3), p = 0.90	-1.8pp (-6.8 to 3.2), p = 0.49
INR variability*	0.03 (-0.18 to 0.24), p = 0.75	-0.34 (-0.57 to -0.12), p = 0.00
B		
TTR	-9.8pp (-12.9 to -6.7), p = 0.00	3.9pp (0.4 to 7.4), p = 0.03
above range	12.3pp (9.2 to 15.4), p = 0.00	1.7pp (-1.3 to 4.7), p = 0.26
below range	-2.5pp (-4.9 to 0.0), p = 0.05	-5.6pp (-8.4 to -2.8), p = 0.00
INR variability*	0.08 (-0.06 to 0.23), p = 0.26	-0.35 (-0.50 to -0.21), p = 0.00
C		
TTR	-5.4pp (-10.8 to 0.0), p = 0.05	6.4pp (0.8 to 12.1), p = 0.03
above range	6.2pp (0.2 to 12.3), p = 0.04	-1.3pp (-7.6 to 4.9), p = 0.68
below range	-0.8pp (-6.3 to 4.6), p = 0.77	-5.1pp (-10.6 to 0.4), p = 0.07
INR variability*	0.14 (-0.06 to 0.34), p = 0.17	-0.55 (-0.76 to -0.34), p = 0.00

An asterisk indicates that values were log-transformed in the analyses.

P-values from a linear mixed model.

#### Effect in subgroups

We compared the effect of a switch in several groups of special interest. Patient characteristics of switchers in these groups, and their matched non-switchers, are summarised in S4 Table. Again, matching was successful: standardised mean differences in propensity scores were <0.1 in all subgroups, except in some subgroups in target range 2.5–3.5: patients with high INR variability (standardised mean difference (SMD) in propensity scores 0.13), elderly patients (SMD 0.20), and patients with mechanical heart valves (SMD 0.12).

**Fig 6:**
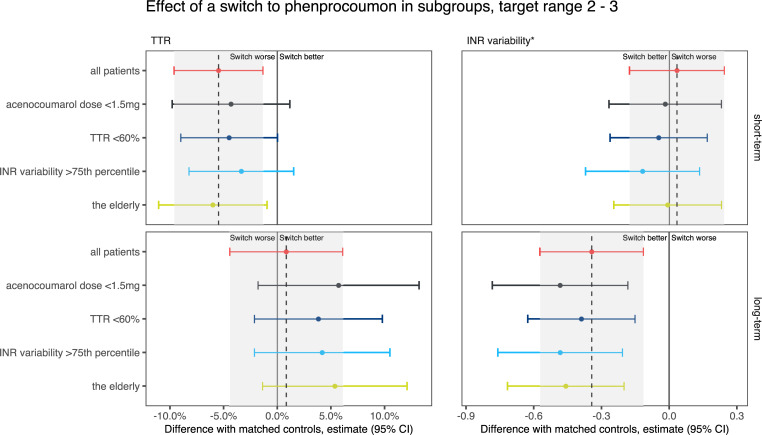
Changes in time in the therapeutic range (TTR) of 2.0–3.0 and international normalised ratio (INR) variability in patients who switched from acenocoumarol to phenprocoumon, relative to matched non-switchers. The grey area indicates the 95% confidence interval of the difference in the overall analysis.

**Fig 7:**
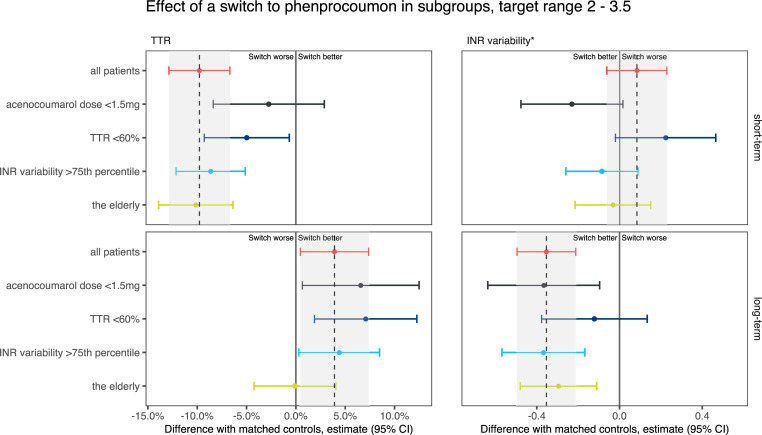
Changes in time in the therapeutic range (TTR) of 2.0–3.5 and international normalised ratio (INR) variability in patients who switched from acenocoumarol to phenprocoumon, relative to matched non-switchers. The grey area indicates the 95% confidence interval of the difference in the overall analysis.

**Fig 8:**
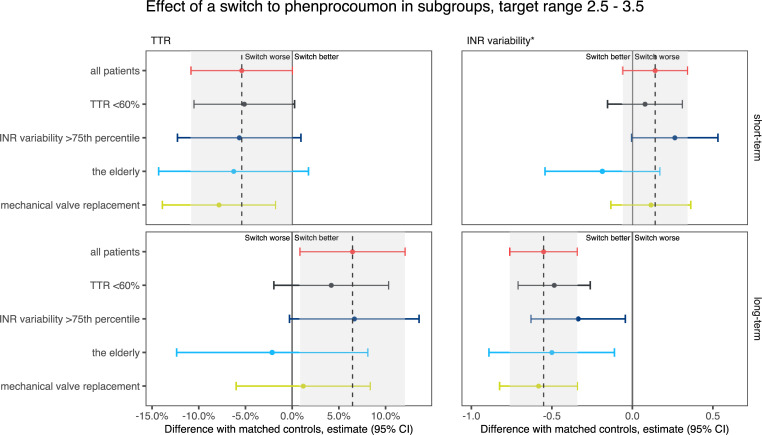
Changes in time in the therapeutic range (TTR) of 2.5–3.5 and international normalised ratio (INR) variability in patients who switched from acenocoumarol to phenprocoumon, relative to matched non-switchers. The grey area indicates the 95% confidence interval of the difference in the overall analysis.

The effect of a switch relative to non-switchers is summarised in S5 Table and shown in Figs 6–8.

The effect of a switch on TTR was consistent in groups with different prompts for switching (a low dose, a poor TTR or a high INR variability). In target ranges 2–3 and 2–3.5, patients with a poor TTR and patients with high INR variability who switched were initially relatively over-anticoagulated compared with patients with a low VKA dose before their switch. This difference disappeared after the first 180 days. In target range 2–3.5, the INRs of patients whose switch was prompted by a low VKA dose stabilised quicker than the INRs of patients with a poor TTR.

We also evaluated the effect of a switch in patients who require specific clinical consideration: i.e. the elderly and patients with a mechanical heart valve. Initially, the effect of a switch on TTR in these groups is comparable to the effect in the whole population. In the long term, however, the TTR of elderly patients in target ranges 2–3.5 and 2.5–3.5 seems not to improve by switching, nor does the TTR of patients with a mechanical heart valve and target range 2.5–3.5.

## Discussion

In this study, we analysed the effect of switching from acenocoumarol to phenprocoumon on the achieved quality of anticoagulation in patients from two Dutch anticoagulation clinics. We found that patients’ time in the therapeutic range and INR variability do not improve in the first six months, compared with non-switching matched controls. In the second six-month period after a switch, however, INR variability decreases and TTR improves.

Although the question which vitamin K antagonist to prefer is far from new, we are the first to observe the real-life effect of switching from acenocoumarol to phenprocoumon using matched non-switchers. Previous research only compared groups of patients using different VKA. Already in the 1960s and early 1970s phenprocoumon showed favourable properties compared with warfarin [8] and acenocoumarol [9,10] on then-used monitoring parameters. After the development of the international normalised ratio (INR) to monitor VKA, several additional studies have been performed, with mixed results. The proportion of INR measurements in range was higher on warfarin than on acenocoumarol in an Italian retrospective cohort study [11], but not in one from Bosnia and Herzegovina [12]. Two retrospective studies from the Netherlands found a higher TTR in patients on phenprocoumon, compared with acenocoumarol [5,13]. In clinical practice, anticoagulation clinics in the Netherlands report higher TTRs in patients on phenprocoumon than patients on acenocoumarol [14]. A retrospective study focussing on self-managed patients found a higher TTR on phenprocoumon than on warfarin [15]. A French randomised controlled trial showed no difference in TTR between patients initiated with warfarin versus those with acenocoumarol, but INR variability was lower in the warfarin group [16]. This implies that phenprocoumon (with the longest half-life) might lead to a higher TTR than warfarin (with the intermediate half-life); warfarin, in turn, could cause a higher or similar TTR to acenocoumarol (with the shortest half-life). However, these studies did not assess the effect of switching from one VKA to another on the quality of anticoagulation.

Two studies describe the switch from acenocoumarol to warfarin. An Italian group found no increase in the number of prothrombin times in range [17]. The results of this study are difficult to generalise because the group of patients had heterogeneous indications for anticoagulation and it is unclear how these patients were selected. A Polish study, confined to younger patients with arbitrarily defined “unstable anticoagulation”, found a marked increase in TTR after the switch [18]. Because both studies lacked a control group, and TTR is not stable over time [19], their results could be distorted by “regression” to the mean.

In our study, we separated the switching effect from the natural course of anticoagulation therapy and bias by indication with the use of matched non-switching controls. However, matching is sensitive to unmeasured confounders. We have included almost all the limited data available to anticoagulation clinics in the matching, and we believe they included the most relevant confounders. However, we could not control for a switch due to the start of interacting medication (e.g. rifampicin).

We could not assess the effect of a switch on the incidence of bleeding and thrombotic events, because we lacked the data. Instead, we measured TTR and INR variability. TTR and INR variability link the joint pharmacological effect of all vitamin K antagonists to bleeding and thrombotic complications [1–3]. Additional mechanisms leading to bleeding or thrombosis, specific to one vitamin K antagonist, are unknown and implausible. Therefore, TTR and INR variability are accepted proxy measures.

The observational nature of our study only allowed us to analyse patients who actually switched. Strictly speaking, our study cannot be generalised to patients whose physicians do not see an indication to switch. However, our results are similar to those from earlier studies comparing patients on the two drugs without switching [5,13]. Nevertheless, the TTR improvement in our study seems smaller than the TTR difference observed between anticoagulation clinics using primarily phenprocoumon instead of acenocoumarol [14]. We have insufficient data to assess whether this effect persists after adjustment for differences in casemix. Another explanation would be a difference in experience with managing phenprocoumon.

Other strong points of our study are the real-life setting and the analysis in contemporary target ranges; previously, higher intensity anticoagulation therapy was prescribed. Our findings are more robust because they stem from two independent thrombosis services.

The eventual increase in TTR and decrease in INR variability after switching to phenprocoumon are intuitive; factor VII levels fluctuate more during the day under acenocoumarol than under phenprocoumon [20,21]. This effect could be more prominent in higher target ranges, where small variances in coagulation factors result in higher changes in the INR than in lower target ranges [22]. However, it is unknown whether the increased stability of factor VII levels on phenprocoumon also extends over periods of weeks instead of a single day. Furthermore, this cannot explain why the beneficial effect is only seen after a “transition period”.

The transition period following a switch is characterised by unfavourable effects on VKA control: a decreased TTR and a higher INR variability. Patients are more prone to bleeding, owing to a higher proportion of time above the target range [2]. A possible cause is the trial-and-error during the determination of the optimal individual phenprocoumon dose. In general, the anticoagulation clinics follow the Dutch guideline, which prescribes a conversion factor of 84% [23]. However, the resulting over-anticoagulation and the later dose reductions suggest that this factor, identified in stable patients in higher target ranges [24], might not be applicable anymore.

In the long run, switching from acenocoumarol to phenprocoumon leads to improved VKA control. In all target ranges, the switch to phenprocoumon significantly decreases INR variability. A lower INR variability reduces the risk of bleeding and thrombosis [25–29], but to what extent is difficult to quantify since different authors use different methods to calculate INR variability. There is no consensus about risk categories based on absolute values of INR variability, and it remains unclear whether INR variability should be interpreted similarly in different target ranges.

A switch to phenprocoumon also significantly improves TTR in target ranges 2–3.5 and 2.5–3.5, relative to non-switching controls. The TTR improvement is most pronounced in target range 2.5–3.5, with a difference of 6.4 (95% CI 0.8 to 12.1) percentage points. The associated clinical benefit cannot be quantified exactly because the relationship between TTR and incidence of events is not linear [2]. Patients with a TTR ≥ 65% have substantially reduced risks of bleeding, thrombosis and death, compared with patients with a TTR < 65% [1]. One additional patient out of every 6.5 (95% CI 4.0–18.1) patients in target range 2.5–3.5 who switch, attains a TTR in this favourable risk category. TTR differences were smaller in target ranges 2–3 and 2–3.5, and more patients need to switch to cause an additional TTR ≥ 65%: 48.0 (95% CI NNT 7.7 to ∞ to NNH 11.3) and 16.9 (95% CI NNT 7.7 to ∞ to NNH 84.5), respectively. One could argue that the effect of moving from a TTR < 65% to a TTR ≥ 65% with a small TTR difference is trivial, because the differences in these categories will be driven by a larger TTR variation.

The benefit of an improved TTR does not extend to patients with a mechanical heart valve prosthesis, nor to elderly patients in target range 2–3.5 or 2.5–3.5 (the latter even seem to decline). Although the findings in this group are less robust, owing to the smaller sample size and less perfect matching, they are cause for concern. The efficacy and safety of VKA are paramount in these groups because alternative forms of anticoagulation might be contraindicated.

Fortunately, elderly patients in target range 2–3 do increase in TTR after the switch. Elderly patients across the target ranges, and patients with mechanical heart valves, obtain a lower INR variability. However, it is questionable whether the decreased INR variability cancels out the neutral or unfavourable effect on TTR, the risks associated with the transition period, and the risk of medication errors with drugs that are less commonly used.

## Conclusion

Eventually, the switch from acenocoumarol to phenprocoumon improves the time in the therapeutic range and decreases the INR variability. However, this is preceded by a transition period, where TTR is lower and mostly the time above the range is increased. Physicians and their patients should weigh the benefit of a small improvement in VKA control and the risk of the transition period.

## Supporting information

S1 Table Patient characteristics from all potential controls. (DOCX)

S2 Table Direct effect of switching in subgroups. See also S6 Table.
(DOCX)

S3 Table Coefficients from logistic regression used in the propensity score matching. (DOCX)

S4 Table Patient characteristics from switchers and selected non-switchers. (DOCX)

S5 Table Patient characteristics from switchers and selected non-switchers in subgroups. (DOCX)

S6 Table Effect of switching from acenocoumarol to phenprocoumon, compared with non-switchers. Estimate with 95% confidence interval. An asterisk indicates that values were log-transformed in the analyses.
(DOCX)
