# Supplementary material for: Effect of switching from acenocoumarol to phenprocoumon on time in therapeutic range and INR variability: A cohort study
Source: PLoS One. 2020 Jul 10;15(7):e0235639. doi: 10.1371/journal.pone.0235639 (PMC7351201; doi:10.1371/journal.pone.0235639)
Supplement: S1 Table — (DOCX) [file pone.0235639.s001.docx]

Supplement to ‘Effect of switching from acenocoumarol to phenprocoumon on time in therapeutic range and INR variability: a cohort study’

**Table S1. Patient characteristics from all potential controls.**

| **Target range** | **2 - 3** | **2 - 3.5** | **2.5 - 3.5** |
| --- | --- | --- | --- |
| N | 8901 | 18683 | 4319 |
| Center Groningen | 7226 | 17259 | 4007 |
| Center Maastricht | 1675 | 1424 | 312 |
| Age (median [IQR]) | 78 [70, 85] | 77 [69, 83] | 74 [65, 81] |
| Male gender (%) | 4536 (51.0) | 9370 (50.2) | 2726 (63.1) |
| VKA experience (median [IQR]) | 3 [1, 6] | 3 [1, 6] | 5 [2, 11] |
| Dose (median [IQR]) | 2.1 [1.5, 2.8] | 2.2 [1.7, 3.0] | 2.3 [1.7, 3.1] |
| Acenocoumarol dose <1.5mg (%) | 2097 (23.8) | 3454 (18.8) | 668 (15.8) |
| Below range (median [IQR]) | 8.3 [0.0, 19.3] | 2.8 [0.0, 11.6] | 12.2 [1.7, 29.8] |
| TTR (median [IQR]) | 69.1 [55.2, 82.9] | 82.3 [69.1, 93.4] | 50.8 [37.0, 66.3] |
| Above range (median [IQR]) | 16.6 [4.4, 30.4] | 8.8 [0.0, 20.4] | 25.4 [8.3, 44.8] |
| TTR <60% (%) | 2930 (32.9) | 2560 (13.7) | 2820 (65.3) |
| Mean INR (median [IQR]) | 2.6 [2.4, 2.8] | 2.8 [2.6, 3.1] | 3.2 [2.8, 3.5] |
| INR variability (median [IQR]) | 0.19 [0.12, 0.30] | 0.22 [0.13, 0.34] | 0.26 [0.17, 0.40] |
| Mean number of days between INRs (median [IQR]) | 19.4 [15.3, 26.8] | 19.3 [14.6, 26.6] | 17.5 [13.8, 23.5] |
| Atrial fibrillation (%) | 7471 (83.9) | 16122 (86.3) | 2921 (67.6) |
| Venous thromboembolism (%) | 1503 (16.9) | 2929 (15.7) | 383 (8.9) |
| Mechanical heart valve (%) | 137 (1.5) | 64 (0.3) | 1591 (36.8) |
