# Supplementary material for: Effect of switching from acenocoumarol to phenprocoumon on time in therapeutic range and INR variability: A cohort study
Source: PLoS One. 2020 Jul 10;15(7):e0235639. doi: 10.1371/journal.pone.0235639 (PMC7351201; doi:10.1371/journal.pone.0235639)
Supplement: S2 Table — See also S6 Table. (DOCX) [file pone.0235639.s002.docx]

Supplement to ‘Effect of switching from acenocoumarol to phenprocoumon on time in therapeutic range and INR variability: a cohort study’

**Table S2. Direct effect of switching in subgroups.**
See also table S6.

| **Target range** | **Subgroup** | **Parameter** | **Before switch** | **Short-term** | **Long-term** |
| --- | --- | --- | --- | --- | --- |
| 2 - 3 | Elderly | dose | 1.0 [0.8 – 1.5] | 0.9 [0.7 – 1.5], p = <0.001 | 0.9 [0.7 – 1.5], p = <0.001 |
|  |  | dose ratio | ref | 92% [82 – 103], p = <0.001 | 88% [75 – 100], p = <0.001 |
|  |  | mean number of days between INRs | 12 [10 – 14] | 11 [10 – 14], p = 0.964 | 17 [14 – 20], p = <0.001 |
|  | Low dose | dose | 0.9 [0.8 – 1.0] | 0.8 [0.7 – 0.9], p = 0.009 | 0.8 [0.7 – 0.9], p = 0.002 |
|  |  | dose ratio | ref | 94% [84 – 105], p = <0.001 | 90% [77 – 103], p = <0.001 |
|  |  | mean number of days between INRs | 12 [10 – 14] | 11 [10 – 13], p = 0.391 | 18 [14 – 21], p = <0.001 |
|  | Poor TTR | dose | 1.1 [0.9 – 2.6] | 1.0 [0.8 – 2.2], p = <0.001 | 1.1 [0.8 – 2.0], p = <0.001 |
|  |  | dose ratio | ref | 92% [79 – 103], p = <0.001 | 87% [70 – 101], p = <0.001 |
|  |  | mean number of days between INRs | 11 [9 – 14] | 11 [10 – 13], p = 0.925 | 18 [13 – 21], p = <0.001 |
|  | Volatile | dose | 1.0 [0.8 – 2.1] | 0.9 [0.8 – 1.7], p = <0.001 | 0.9 [0.7 – 1.6], p = <0.001 |
|  |  | dose ratio | ref | 91% [78 – 103], p = <0.001 | 85% [73 – 99], p = <0.001 |
|  |  | mean number of days between INRs | 11 [9 – 13] | 11 [10 – 13], p = 0.863 | 17 [13 – 21], p = <0.001 |
| 2 - 3.5 | Elderly | dose | 2.1 [0.9 – 3.1] | 1.8 [0.9 – 2.6], p = <0.001 | 1.6 [0.9 – 2.3], p = <0.001 |
|  |  | dose ratio | ref | 89% [76 – 101], p = <0.001 | 81% [68 – 92], p = <0.001 |
|  |  | mean number of days between INRs | 14 [11 – 18] | 12 [11 – 14], p = <0.001 | 18 [14 – 23], p = <0.001 |
|  | Low dose | dose | 0.9 [0.8 – 1.0] | 0.8 [0.7 – 1.1], p = 0.741 | 0.8 [0.6 – 1.0], p = 0.032 |
|  |  | dose ratio | ref | 96% [86 – 114], p = <0.001 | 92% [79 – 110], p = <0.001 |
|  |  | mean number of days between INRs | 12 [10 – 15] | 12 [10 – 14], p = 0.049 | 19 [13 – 22], p = <0.001 |
|  | Poor TTR | dose | 2.8 [1.0 – 4.3] | 2.2 [1.1 – 3.3], p = <0.001 | 2.0 [1.0 – 3.1], p = <0.001 |
|  |  | dose ratio | ref | 83% [70 – 101], p = <0.001 | 78% [64 – 94], p = <0.001 |
|  |  | mean number of days between INRs | 11 [10 – 14] | 12 [9 – 13], p = 0.555 | 16 [12 – 22], p = <0.001 |
| 2 - 3.5 | Volatile | dose | 2.3 [0.9 – 3.4] | 1.8 [0.9 – 2.9], p = <0.001 | 1.8 [0.9 – 2.6], p = <0.001 |
| (cont) |  | dose ratio | ref | 88% [76 – 101], p = <0.001 | 82% [69 – 96], p = <0.001 |
|  |  | mean number of days between INRs | 12 [10 – 14] | 12 [9 – 14], p = 0.102 | 17 [13 – 22], p = <0.001 |
| 2.5 - 3.5 | Elderly | dose | 1.8 [0.9 – 3.3] | 1.7 [1.0 – 2.5], p = <0.001 | 1.6 [0.8 – 2.5], p = <0.001 |
|  |  | dose ratio | ref | 91% [78 – 99], p = <0.001 | 80% [66 – 92], p = <0.001 |
|  |  | mean number of days between INRs | 11 [9 – 13] | 11 [9 – 12], p = 0.227 | 15 [11 – 18], p = 0.002 |
|  | Poor TTR | dose | 3.1 [1.7 – 4.3] | 2.5 [1.6 – 3.5], p = <0.001 | 2.2 [1.6 – 3.3], p = <0.001 |
|  |  | dose ratio | ref | 88% [73 – 98], p = <0.001 | 81% [68 – 94], p = <0.001 |
|  |  | mean number of days between INRs | 12 [9 – 14] | 11 [9 – 13], p = 0.100 | 16 [11 – 20], p = <0.001 |
|  | Valve | dose | 3.3 [2.2 – 4.3] | 2.6 [1.8 – 3.6], p = <0.001 | 2.4 [1.7 – 3.4], p = <0.001 |
|  |  | dose ratio | ref | 85% [72 – 94], p = <0.001 | 78% [66 – 90], p = <0.001 |
|  |  | mean number of days between INRs | 11 [9 – 13] | 11 [9 – 13], p = 0.074 | 16 [13 – 21], p = <0.001 |
|  | Volatile | dose | 3.2 [1.4 – 4.2] | 2.4 [1.5 – 3.5], p = <0.001 | 2.4 [1.5 – 3.3], p = <0.001 |
|  |  | dose ratio | ref | 88% [71 – 98], p = <0.001 | 79% [64 – 92], p = <0.001 |
|  |  | mean number of days between INRs | 10 [8 – 12] | 10 [8 – 12], p = 0.311 | 14 [11 – 18], p = <0.001 |
