# Supplementary material for: Effect of switching from acenocoumarol to phenprocoumon on time in therapeutic range and INR variability: A cohort study
Source: PLoS One. 2020 Jul 10;15(7):e0235639. doi: 10.1371/journal.pone.0235639 (PMC7351201; doi:10.1371/journal.pone.0235639)
Supplement: S3 Table — (DOCX) [file pone.0235639.s003.docx]

Supplement to ‘Effect of switching from acenocoumarol to phenprocoumon on time in therapeutic range and INR variability: a cohort study’

**Table S3. Coefficients from logistic regression used in the propensity score matching.**

|  |  | Subgroup | | | | | |
| --- | --- | --- | --- | --- | --- | --- | --- |
| Target Range | term | all | elderly | low dose | poor TTR | valve | volatile |
| 2-3 | (Intercept) | 0.01 | 0.01 | 0.06 | 0.05 | NA | 0.01 |
|  | VKA_exp | 1.08 | 1.09 | 1.09 | 1.09 | NA | 1.09 |
|  | above_range | 12.51 | 18.49 | 16.20 | 1.67 | NA | 20.25 |
|  | age | 0.98 | 0.99 | 0.98 | 0.98 | NA | 0.99 |
|  | below_range | 200.70 | 155.64 | 42.40 | 25.01 | NA | 65.26 |
|  | dose_cathigh | 5.37 | 3.92 | NA | 6.26 | NA | 4.23 |
|  | dose_catlow | 2.64 | 2.46 | NA | 3.02 | NA | 2.47 |
|  | genderMale | 0.95 | 0.71 | 0.81 | 0.98 | NA | 0.95 |
|  | ind_MVRTRUE | 1.72 | 0.95 | 0.32 | 1.63 | NA | 1.67 |
|  | ind_VTETRUE | 0.89 | 0.76 | 0.65 | 0.91 | NA | 0.86 |
|  | log(vgr) | 3.39 | 3.22 | 2.95 | 3.56 | NA | 2.52 |
| 2-3.5 | (Intercept) | 0.14 | 1.09 | 0.07 | 0.05 | NA | 0.10 |
|  | VKA_exp | 1.02 | 1.02 | 1.07 | 1.04 | NA | 1.05 |
|  | above_range | 3.00 | 7.77 | 19.66 | 6.14 | NA | 4.58 |
|  | age | 0.97 | 0.95 | 0.98 | 0.98 | NA | 0.97 |
|  | below_range | 62.40 | 41.70 | 64.87 | 92.25 | NA | 67.08 |
|  | dose_cathigh | 2.67 | 2.16 | NA | 2.64 | NA | 1.78 |
|  | dose_catlow | 0.93 | 0.89 | NA | 1.00 | NA | 1.15 |
|  | genderMale | 0.71 | 0.71 | 1.25 | 0.83 | NA | 0.78 |
|  | ind_MVRTRUE | 2.79 | 0.00 | 0.00 | 2.76 | NA | 2.22 |
|  | ind_VTETRUE | 0.80 | 0.70 | 0.60 | 0.83 | NA | 0.81 |
|  | log(vgr) | 2.36 | 2.54 | 3.16 | 2.45 | NA | 2.23 |
| 2.5-3.5 | (Intercept) | 0.06 | 0.04 | NA | 0.07 | 0.11 | 0.07 |
|  | VKA_exp | 1.02 | 1.07 | NA | 1.02 | 1.01 | 1.02 |
|  | above_range | 6.92 | 7.86 | NA | 10.85 | 4.95 | 8.39 |
|  | age | 0.97 | 0.96 | NA | 0.97 | 0.99 | 0.97 |
|  | below_range | 68.90 | 133.54 | NA | 112.28 | 34.48 | 46.81 |
|  | dose_cathigh | 1.89 | 2.60 | NA | 1.88 | 1.74 | 1.63 |
|  | dose_catlow | 0.91 | 1.26 | NA | 0.90 | 0.52 | 0.76 |
|  | genderMale | 0.71 | 0.84 | NA | 0.67 | 0.68 | 0.49 |
|  | ind_MVRTRUE | 2.82 | 3.01 | NA | 2.51 | NA | 3.51 |
|  | ind_VTETRUE | 1.67 | 0.00 | NA | 1.80 | 0.00 | 1.61 |
|  | log(vgr) | 3.07 | 4.31 | NA | 3.36 | 3.73 | 3.34 |
