# Supplementary material for: Effect of switching from acenocoumarol to phenprocoumon on time in therapeutic range and INR variability: A cohort study
Source: PLoS One. 2020 Jul 10;15(7):e0235639. doi: 10.1371/journal.pone.0235639 (PMC7351201; doi:10.1371/journal.pone.0235639)
Supplement: S4 Table — (DOCX) [file pone.0235639.s004.docx]

Supplement to ‘Effect of switching from acenocoumarol to phenprocoumon on time in therapeutic range and INR variability: a cohort study’

**Table S4. Patient characteristics from switchers and selected non-switchers.**

|  | 2 - 3 | | 2 – 3.5 | | 2.5 – 3.5 | |
| --- | --- | --- | --- | --- | --- | --- |
|  | switchers | non-switchers | switchers | non-switchers | switchers | non-switchers |
| N | 124 | 248 | 269 | 538 | 98 | 196 |
| Center Groningen | 30 | 79 | 163 | 151 | 66 | 89 |
| Center Maastricht | 94 | 169 | 106 | 387 | 32 | 107 |
| Age (median [IQR]) | 80 [69, 85] | 81 [73, 87] | 73 [62, 80] | 75 [65, 82] | 65 [56, 74] | 67 [55, 76] |
| Male gender (%) | 56 (45.2) | 108 (43.5) | 121 (45.0) | 257 (47.8) | 53 (54.1) | 109 (55.6) |
| VKA experience (median [IQR]) | 5 [3, 11] | 5 [2, 12] | 2 [1, 5] | 3 [1, 7] | 5 [2, 12] | 7 [2, 13] |
| Dose (median [IQR]) | 1.1 [0.9, 2.8] | 1.5 [1.1, 2.0] | 2.7 [1.5, 4.3] | 2.6 [1.8, 4.0] | 3.1 [1.8, 4.3] | 3.0 [2.1, 4.3] |
| Acenocoumarol dose <1.5mg (%) | 75 (60.5) | 123 (49.6) | 68 (25.3) | 79 (14.7) | 21 (21.4) | 16 (8.2) |
| Below range (median [IQR]) | 23.8 [15.5, 37.7] | 24.9 [14.4, 38.3] | 13.8 [5.0, 27.6] | 13.3 [3.3, 26.5] | 28.5 [10.5, 49.0] | 22.1 [7.2, 43.2] |
| TTR (median [IQR]) | 45.3 [33.6, 53.7] | 47.5 [34.1, 59.7] | 64.1 [47.0, 79.0] | 62.4 [50.3, 75.1] | 35.6 [21.7, 47.2] | 38.0 [26.0, 51.0] |
| Above range (median [IQR]) | 27.9 [15.7, 40.5] | 23.2 [12.2, 36.0] | 14.4 [2.2, 29.8] | 18.8 [8.3, 29.3] | 32.3 [13.3, 46.8] | 31.5 [16.0, 45.3] |
| TTR <60% (%) | 106 (85.5) | 189 (76.2) | 117 (43.5) | 240 (44.6) | 86 (87.8) | 175 (89.3) |
| Mean INR (median [IQR]) | 2.7 [2.3, 2.9] | 2.7 [2.4, 2.9] | 2.8 [2.4, 3.2] | 2.9 [2.6, 3.2] | 3.2 [2.7, 3.6] | 3.2 [2.8, 3.5] |
| INR variability (median [IQR]) | 0.52 [0.32, 0.80] | 0.48 [0.33, 0.85] | 0.40 [0.23, 0.67] | 0.43 [0.28, 0.67] | 0.50 [0.31, 0.93] | 0.49 [0.32, 0.76] |
| Mean number of days between INRs (median [IQR]) | 11.7 [9.6, 14.6] | 12.4 [10.3, 15.3] | 14.0 [10.7, 17.5] | 14.4 [11.4, 18.4] | 12.0 [9.1, 14.6] | 13.3 [11.2, 16.1] |
| Atrial fibrillation (%) | 96 (77.4) | 196 (79.0) | 220 (81.8) | 455 (84.6) | 42 (42.9) | 65 (33.2) |
| Venous thromboembolism (%) | 27 (21.8) | 47 (19.0) | 52 (19.3) | 85 (15.8) | 11 (11.2) | 13 (6.6) |
| Mechanical heart valve (%) | 6 (4.8) | 10 (4.0) | 4 (1.5) | 9 (1.7) | 63 (64.3) | 152 (77.6) |
