# Supplementary material for: Effect of switching from acenocoumarol to phenprocoumon on time in therapeutic range and INR variability: A cohort study
Source: PLoS One. 2020 Jul 10;15(7):e0235639. doi: 10.1371/journal.pone.0235639 (PMC7351201; doi:10.1371/journal.pone.0235639)
Supplement: S5 Table — (DOCX) [file pone.0235639.s005.docx]

Supplement to ‘Effect of switching from acenocoumarol to phenprocoumon on time in therapeutic range and INR variability: a cohort study’

| Target range 2.0 – 3.0 | Elderly | | Low dose | | Poor TTR | | Volatile | |
| --- | --- | --- | --- | --- | --- | --- | --- | --- |
|  | switchers | non-switchers | switchers | non-switchers | switchers | non-switchers | switchers | non-switchers |
| N | 89 | 178 | 75 | 150 | 106 | 212 | 97 | 194 |
| Center Groningen | 7 | 44 | 4 | 68 | 24 | 72 | 16 | 56 |
| Center Maastricht | 82 | 134 | 71 | 82 | 82 | 140 | 81 | 138 |
| Age (median [IQR]) | 82 [79, 87] | 84 [80, 88] | 82 [78, 87.5] | 84 [80, 90] | 81 [69, 86] | 82 [72.8, 87] | 81 [75, 87] | 83 [76, 88.8] |
| Male gender (%) | 31 (34.8) | 62 (34.8) | 29 (38.7) | 53 (35.3) | 47 (44.3) | 91 (42.9) | 42 (43.3) | 84 (43.3) |
| VKA experience (median [IQR]) | 6 [3, 11] | 6 [2, 11.8] | 6 [3, 11] | 5 [2, 10] | 5 [3, 11] | 5 [2, 11] | 6 [3, 11] | 6 [2, 12] |
| Dose (median [IQR]) | 1.0 [0.8, 1.5] | 1.5 [1.1, 1.8] | 0.9 [0.8, 1] | 1.2 [0.9, 1.4] | 1.1 [0.9, 2.6] | 1.6 [1.2, 2.1] | 1.0 [0.8, 2.1] | 1.5 [1.2, 2] |
| Acenocoumarol dose <1.5mg (%) | 67 (75.3) | 92 (51.7) | 75 (100) | 150 (100) | 68 (64.2) | 89 (42) | 68 (70.1) | 94 (48.5) |
| Below range (median [IQR]) | 23.2 [16, 33.7] | 22.9 [13.3, 35.8] | 21.8 [14.9, 28.7] | 22.1 [9.5, 31.5] | 24.9 [16.2, 39.6] | 30.7 [19.3, 39.8] | 21 [14.9, 31.5] | 22.7 [12.7, 36.5] |
| TTR (median [IQR]) | 45.3 [34.3, 51.9] | 48.3 [36.3, 58.4] | 47 [36.1, 51.7] | 47 [32.7, 58.4] | 42 [31.9, 50.3] | 45.3 [34.8, 53.7] | 43.6 [33.1, 50.8] | 45.0 [33.1, 56.8] |
| Above range (median [IQR]) | 29.8 [19.9, 41.4] | 26 [15.1, 37.4] | 30.4 [21.5, 42.8] | 29.3 [17.1, 43] | 30.7 [20.5, 42.3] | 25.1 [15.5, 35.4] | 33.7 [23.8, 42.5] | 27.1 [17.7, 39.6] |
| TTR <60% (%) | 77 (86.5) | 141 (79.2) | 68 (90.7) | 118 (78.7) | 106 (100) | 212 (100) | 90 (92.8) | 159 (82) |
| Mean INR (median [IQR]) | 2.7 [2.4, 2.9] | 2.7 [2.4, 3] | 2.8 [2.6, 3] | 2.8 [2.5, 3.1] | 2.7 [2.4, 3] | 2.7 [2.3, 3] | 2.8 [2.6, 3.1] | 2.7 [2.5, 3.1] |
| INR variability (median [IQR]) | 0.55 [0.39, 0.79] | 0.54 [0.35, 13] | 0.57 [0.43, 0.84] | 0.61 [0.38, 1.25] | 0.56 [0.39, 11] | 0.56 [0.37, 11] | 0.60 [0.45, 14] | 0.59 [0.40, 1.12] |
| Mean number of days between INRs (median [IQR]) | 11.7 [9.7, 14.3] | 12 [10.3, 14.7] | 12 [10, 14.4] | 12.4 [10.5, 15.1] | 11.1 [9.3, 13.8] | 11.7 [9.9, 13.5] | 11.1 [9.3, 13.5] | 12 [10.1, 13.7] |
| Atrial fibrillation (%) | 78 (87.6) | 159 (89.3) | 68 (90.7) | 138 (92) | 82 (77.4) | 169 (79.7) | 79 (81.4) | 160 (82.5) |
| Venous thromboembolism (%) | 11 (12.4) | 22 (12.4) | 8 (10.7) | 15 (10) | 23 (21.7) | 38 (17.9) | 18 (18.6) | 32 (16.5) |
| Mechanical heart valve (%) | 3 (3.4) | 1 (0.6) | 1 (1.3) | 1 (0.7) | 5 (4.7) | 9 (4.2) | 5 (5.2) | 6 (3.1) |

|  |  |  |  |  |  |  |  |  |
| --- | --- | --- | --- | --- | --- | --- | --- | --- |
| Target range 2.0 – 3.5 | Elderly | | Low dose | | Poor TTR | | Volatile | |
|  | switchers | non-switchers | switchers | non-switchers | switchers | non-switchers | switchers | non-switchers |
| N | 158 | 316 | 68 | 136 | 117 | 234 | 156 | 312 |
| Center Groningen | 76 | 56 | 18 | 60 | 56 | 116 | 70 | 94 |
| Center Maastricht | 82 | 260 | 50 | 76 | 61 | 118 | 86 | 218 |
| Age (median [IQR]) | 79 [75, 83] | 80 [75, 85] | 82.5 [75.8, 86] | 81 [76, 86] | 73 [62, 83] | 77.5 [67, 85] | 76 [64, 83] | 76 [66, 83] |
| Male gender (%) | 61 (38.6) | 130 (41.1) | 31 (45.6) | 70 (51.5) | 55 (47) | 98 (41.9) | 69 (44.2) | 155 (49.7) |
| VKA experience (median [IQR]) | 3 [1, 6] | 4 [1, 8] | 3 [1, 7] | 4 [1, 9] | 3 [1, 6] | 3 [1, 7.8] | 3 [1, 6] | 3 [1, 7] |
| Dose (median [IQR]) | 2.1 [0.9, 3.1] | 2.1 [1.6, 2.9] | 0.9 [0.8, 1] | 1.2 [0.9, 1.4] | 2.8 [1, 4.3] | 2.4 [1.6, 3.9] | 2.3 [0.9, 3.4] | 2 [1.6, 3.2] |
| Acenocoumarol dose <1.5mg (%) | 58 (36.7) | 66 (20.9) | 68 (100) | 136 (100) | 37 (31.6) | 51 (21.8) | 62 (39.7) | 69 (22.1) |
| Below range (median [IQR]) | 12.4 [5.3, 23.8] | 11.9 [2.8, 19.9] | 16.6 [9.1, 23.9] | 13.3 [5.4, 21] | 27.6 [14.4, 51.9] | 23.6 [11.7, 41.4] | 16 [8.7, 26.5] | 15.2 [7.2, 28.9] |
| TTR (median [IQR]) | 63.5 [47, 78.2] | 63.5 [51.4, 74.7] | 58.6 [46.5, 68.6] | 58.3 [48.6, 68.6] | 44.8 [34.3, 52.5] | 46.7 [36.8, 53.6] | 56.4 [43.5, 69.3] | 56.9 [47, 66.9] |
| Above range (median [IQR]) | 18.5 [6.8, 32] | 22.4 [11.6, 31.1] | 25.7 [15.3, 32.2] | 24 [15.5, 34.8] | 27.1 [7.7, 39.8] | 26.5 [14, 39.4] | 22.1 [12.6, 32.7] | 22.9 [14.2, 33.3] |
| TTR <60% (%) | 70 (44.3) | 136 (43) | 37 (54.4) | 71 (52.2) | 117 (100) | 234 (100) | 87 (55.8) | 183 (58.7) |
| Mean INR (median [IQR]) | 2.9 [2.5, 3.3] | 3 [2.7, 3.3] | 3 [2.7, 3.3] | 3 [2.8, 3.2] | 2.9 [2.1, 3.3] | 2.9 [2.6, 3.3] | 2.9 [2.6, 3.3] | 3 [2.7, 3.3] |
| INR variability (median [IQR]) | 0.45 [0.25, 0.77] | 0.46 [0.30, 0.73] | 0.66 [0.43, 0.96] | 0.63 [0.44, 15] | 0.63 [0.34, 0.92] | 0.65 [0.42, 1.20] | 0.61 [0.46, 0.89] | 0.61 [0.45, 0.98] |
| Mean number of days between INRs (median [IQR]) | 14 [10.7, 17.6] | 14.1 [11.5, 18.4] | 12.5 [10.3, 15] | 12.1 [10.3, 15.4] | 11.1 [9.5, 14] | 11.4 [9.7, 13.5] | 11.9 [9.8, 14.5] | 12.1 [10.3, 14.7] |
| Atrial fibrillation (%) | 149 (94.3) | 296 (93.7) | 63 (92.6) | 130 (95.6) | 96 (82.1) | 192 (82.1) | 131 (84) | 273 (87.5) |
| Venous thromboembolism (%) | 15 (9.5) | 25 (7.9) | 7 (10.3) | 10 (7.4) | 23 (19.7) | 40 (17.1) | 30 (19.2) | 42 (13.5) |
| Mechanical heart valve (%) | NA | NA | NA | NA | 2 (1.7) | 6 (2.6) | 2 (1.3) | 5 (1.6) |

|  |  |  |  |  |  |  |  |  |
| --- | --- | --- | --- | --- | --- | --- | --- | --- |
| Target range 2.5 – 3.5 | Elderly | | Low dose | | Poor TTR | | Volatile | |
|  | switchers | non-switchers | switchers | non-switchers | switchers | non-switchers | switchers | non-switchers |
| N | 37 | 74 | 86 | 172 | 63 | 126 | 63 | 126 |
| Center Groningen | 17 | 31 | 56 | 86 | 39 | 67 | 36 | 84 |
| Center Maastricht | 20 | 43 | 30 | 86 | 24 | 59 | 27 | 42 |
| Age (median [IQR]) | 77 [74, 84] | 78 [74.2, 82] | 65 [52.5, 74] | 67 [56, 75.2] | 65 [56.5, 74] | 67 [56.5, 77] | 65 [51, 74.5] | 67 [55.5, 76.8] |
| Male gender (%) | 19 (51.4) | 40 (54.1) | 45 (52.3) | 96 (55.8) | 33 (52.4) | 68 (54) | 28 (44.4) | 69 (54.8) |
| VKA experience (median [IQR]) | 10 [6, 16] | 11 [6, 19.8] | 5 [2, 12] | 8 [2, 14] | 7 [2, 12] | 6.5 [2, 11] | 7 [2, 12.5] | 6 [2, 14] |
| Dose (median [IQR]) | 1.8 [0.9, 3.3] | 1.9 [1.5, 3] | 3.1 [1.7, 4.3] | 2.9 [1.8, 4.1] | 3.3 [2.2, 4.3] | 3 [2.2, 4.3] | 3.2 [1.4, 4.2] | 2.6 [1.9, 4.1] |
| Acenocoumarol dose <1.5mg (%) | 15 (40.5) | 19 (25.7) | 20 (23.3) | 23 (13.4) | 10 (15.9) | 8 (6.3) | 16 (25.4) | 17 (13.5) |
| Below range (median [IQR]) | 32.6 [14.4, 50.3] | 21.8 [5.8, 38.4] | 32.3 [11.7, 50.8] | 28.3 [11.1, 48.5] | 17.1 [8.3, 35.9] | 17.7 [5.7, 35.9] | 28.2 [12.2, 39.2] | 21.8 [9.3, 38.5] |
| TTR (median [IQR]) | 29.8 [21.5, 42.5] | 33.4 [22.8, 43.6] | 31.2 [21.1, 42.5] | 34 [23.6, 43.2] | 37.6 [25.7, 51.4] | 39.2 [29.3, 49.2] | 32 [21.8, 43.9] | 34.8 [25.1, 46.3] |
| Above range (median [IQR]) | 35.9 [22.1, 47.5] | 36.7 [19.6, 53.2] | 35.4 [13.8, 47.9] | 34.3 [17.7, 48.3] | 38.1 [23.8, 48.3] | 38.1 [20.7, 49.6] | 37 [24.9, 47.5] | 37.8 [23.2, 47.9] |
| TTR <60% (%) | 35 (94.6) | 69 (93.2) | 86 (100) | 172 (100) | 53 (84.1) | 114 (90.5) | 59 (93.7) | 119 (94.4) |
| Mean INR (median [IQR]) | 3.3 [3, 3.6] | 3.3 [2.9, 3.6] | 3.2 [2.6, 3.7] | 3.2 [2.8, 3.5] | 3.4 [3.1, 3.7] | 3.3 [2.9, 3.6] | 3.4 [3.1, 3.7] | 3.3 [3, 3.6] |
| INR variability (median [IQR]) | 0.66 [0.40, 1.17] | 0.54 [0.32, 0.90] | 0.55 [0.37, 0.95] | 0.54 [0.36, 0.92] | 0.58 [0.39, 1.11] | 0.57 [0.37, 0.90] | 0.76 [0.51, 1.24] | 0.73 [0.56, 1.24] |
| Mean number of days between INRs (median [IQR]) | 10.7 [9, 12.8] | 12.6 [10.6, 17.1] | 11.9 [9.1, 14.1] | 12.9 [10.7, 15.9] | 11.2 [8.8, 13.5] | 12.9 [10.9, 16] | 10.3 [8.5, 12.5] | 11.5 [9.6, 13.8] |
| Atrial fibrillation (%) | 25 (67.6) | 32 (43.2) | 39 (45.3) | 64 (37.2) | 17 (27) | 26 (20.6) | 23 (36.5) | 38 (30.2) |
| Mechanical heart valve (%) | 22 (59.5) | 56 (75.7) | 53 (61.6) | 115 (66.9) | 63 (100) | 126 (100) | 46 (73) | 101 (80.2) |
| Venous thromboembolism (%) | NA | NA | 11 (12.8) | 18 (10.5) | NA | NA | 6 (9.5) | 8 (6.3) |

**Table S5. Patient characteristics from switchers and selected non-switchers in subgroups.**
