# Supplementary material for: Effect of switching from acenocoumarol to phenprocoumon on time in therapeutic range and INR variability: A cohort study
Source: PLoS One. 2020 Jul 10;15(7):e0235639. doi: 10.1371/journal.pone.0235639 (PMC7351201; doi:10.1371/journal.pone.0235639)
Supplement: S6 Table — Estimate with 95% confidence interval. An asterisk indicates that values were log-transformed in the analyses. (DOCX) [file pone.0235639.s006.docx]

Supplement to ‘Effect of switching from acenocoumarol to phenprocoumon on time in therapeutic range and INR variability: a cohort study’

**Table S6. Effect of switching from acenocoumarol to phenprocoumon, compared with non-switchers.**
Estimate with 95% confidence interval.
An asterisk indicates that values were log-transformed in the analyses.

| Target range | Subgroup | Variable | Short-term difference | Long-term difference |
| --- | --- | --- | --- | --- |
| 2 - 3 | elderly | TTR | -6.00 (-11.05 to -0.94) | 5.36 (-1.37 to 12.10) |
|  |  | above range | 4.13 (-1.51 to 9.76) | -2.32 (-9.09 to 4.45) |
|  |  | below range | 1.87 (-3.38 to 7.12) | -3.07 (-9.10 to 2.96) |
|  |  | INR variability* | -0.01 (-0.25 to 0.23) | -0.46 (-0.72 to -0.20) |
|  | low dose | TTR | -4.31 (-9.79 to 1.18) | 5.71 (-1.80 to 13.22) |
|  |  | above range | 0.02 (-5.56 to 5.60) | -4.56 (-12.69 to 3.56) |
|  |  | below range | 4.29 (-1.13 to 9.71) | -1.13 (-7.39 to 5.13) |
|  |  | INR variability* | -0.02 (-0.27 to 0.23) | -0.48 (-0.78 to -0.18) |
|  | poor TTR | TTR | -4.48 (-8.99 to 0.02) | 3.83 (-2.13 to 9.79) |
|  |  | above range | 2.60 (-2.41 to 7.60) | -3.77 (-9.49 to 1.96) |
|  |  | below range | 1.89 (-3.07 to 6.84) | -0.06 (-5.49 to 5.38) |
|  |  | INR variability* | -0.05 (-0.26 to 0.17) | -0.39 (-0.63 to -0.15) |
|  | volatile | TTR | -3.34 (-8.22 to 1.54) | 4.18 (-2.13 to 10.50) |
|  |  | above range | 0.08 (-5.06 to 5.21) | -3.56 (-10.32 to 3.21) |
|  |  | below range | 3.27 (-1.66 to 8.19) | -0.60 (-5.99 to 4.79) |
|  |  | INR variability* | -0.12 (-0.37 to 0.13) | -0.48 (-0.76 to -0.21) |
| 2 - 3.5 | elderly | TTR | -10.15 (-13.91 to -6.38) | -0.10 (-4.24 to 4.04) |
|  |  | above range | 10.91 (7.02 to 14.80) | 3.10 (-0.85 to 7.04) |
|  |  | below range | -0.76 (-3.81 to 2.28) | -3.01 (-6.04 to 0.02) |
|  |  | INR variability* | -0.03 (-0.22 to 0.15) | -0.30 (-0.48 to -0.11) |
|  | low dose | TTR | -2.75 (-8.39 to 2.88) | 6.56 (0.64 to 12.48) |
|  |  | above range | 3.00 (-2.64 to 8.65) | 2.13 (-3.20 to 7.46) |
|  |  | below range | -0.25 (-5.28 to 4.78) | -8.59 (-13.25 to -3.94) |
|  |  | INR variability* | -0.23 (-0.48 to 0.02) | -0.37 (-0.64 to -0.10) |
|  | poor TTR | TTR | -4.98 (-9.30 to -0.67) | 7.07 (1.87 to 12.26) |
|  |  | above range | 11.98 (7.27 to 16.68) | 4.84 (-0.23 to 9.91) |
|  |  | below range | -7.00 (-11.64 to -2.35) | -11.92 (-17.26 to -6.59) |
|  |  | INR variability* | 0.22 (-0.02 to 0.47) | -0.12 (-0.38 to 0.13) |
|  | volatile | TTR | -8.64 (-12.15 to -5.13) | 4.39 (0.31 to 8.47) |
|  |  | above range | 8.19 (4.68 to 11.71) | -0.85 (-4.85 to 3.15) |
|  |  | below range | 0.45 (-2.63 to 3.54) | -3.55 (-6.89 to -0.21) |
|  |  | INR variability* | -0.09 (-0.26 to 0.09) | -0.37 (-0.57 to -0.17) |

| Target range | Subgroup | Variable | Short-term difference | Long-term difference |
| --- | --- | --- | --- | --- |
| 2.5 - 3.5 | elderly | TTR | -6.27 (-14.29 to 1.75) | -2.14 (-12.37 to 8.09) |
|  |  | above range | 10.24 (0.24 to 20.24) | 9.66 (-0.87 to 20.19) |
|  |  | below range | -3.97 (-12.87 to 4.93) | -7.52 (-16.33 to 1.29) |
|  |  | INR variability* | -0.19 (-0.54 to 0.17) | -0.50 (-0.89 to -0.11) |
|  | poor TTR | TTR | -5.13 (-10.52 to 0.26) | 4.18 (-1.96 to 10.31) |
|  |  | above range | 7.84 (1.86 to 13.83) | 0.26 (-6.26 to 6.77) |
|  |  | below range | -2.71 (-8.57 to 3.15) | -4.49 (-10.32 to 1.33) |
|  |  | INR variability* | 0.08 (-0.15 to 0.31) | -0.49 (-0.71 to -0.26) |
|  | valve | TTR | -7.84 (-13.89 to -1.80) | 1.18 (-5.99 to 8.35) |
|  |  | above range | 7.21 (-0.25 to 14.67) | 0.40 (-8.00 to 8.80) |
|  |  | below range | 0.63 (-5.19 to 6.45) | -1.56 (-7.99 to 4.86) |
|  |  | INR variability* | 0.11 (-0.13 to 0.36) | -0.58 (-0.82 to -0.34) |
|  | volatile | TTR | -5.67 (-12.28 to 0.94) | 6.65 (-0.29 to 13.59) |
|  |  | above range | 7.67 (0.60 to 14.74) | -0.79 (-8.80 to 7.22) |
|  |  | below range | -2.00 (-7.99 to 3.99) | -5.88 (-12.60 to 0.84) |
|  |  | INR variability* | 0.26 (-0.01 to 0.53) | -0.34 (-0.63 to -0.04) |

Table S6. Effect of switching from acenocoumarol to phenprocoumon, compared with non-switchers.
Estimate with 95% confidence interval.
An asterisk indicates that values were log-transformed in the analyses.
